# Lung Biomolecular Profile and Function of Grafts from Donors after Cardiocirculatory Death with Prolonged Donor Warm Ischemia Time

**DOI:** 10.3390/jcm11113066

**Published:** 2022-05-29

**Authors:** Francesca Gori, Jacopo Fumagalli, Caterina Lonati, Andrea Carlin, Patrizia Leonardi, Osvaldo Biancolilli, Antonello Rossetti, Ilaria Righi, Davide Tosi, Alessandro Palleschi, Lorenzo Rosso, Letizia Corinna Morlacchi, Francesco Blasi, Luigi Vivona, Gaetano Florio, Vittorio Scaravilli, Franco Valenza, Alberto Zanella, Giacomo Grasselli

**Affiliations:** 1 Department of Anesthesia, Critical Care and Emergency, Fondazione IRCCS Ca’ Granda-Ospedale Maggiore Policlinico, 20122 Milan, Italy; francesca.gori3@libero.it (F.G.); jacopo.fumagalli@live.it (J.F.); osvaldo.biancolilli85@gmail.com (O.B.); vittorio.scaravilli@gmail.com (V.S.); giacomo.grasselli@unimi.it (G.G.); 2 Center of Preclinical Research, Fondazione IRCCS Ca’ Granda-Ospedale Maggiore Policlinico, 20122 Milan, Italy; caterina.lonati@gmail.com; 3 Department of Pathophysiology and Transplantation, University of Milan, 20122 Milan, Italy; andrea.carlin@unimi.it (A.C.); patrizia.leonardi@unimi.it (P.L.); alepalleschi@gmail.com (A.P.); lorenzorosso1@gmail.com (L.R.); francesco.blasi@unimi.it (F.B.); luigi.vivona@unimi.it (L.V.); g.floriomed@gmail.com (G.F.); franco.valenza@gmail.com (F.V.); 4 Hospital Medical Direction, Fondazione IRCCS Ca’ Granda-Ospedale Maggiore Policlinico, 20122 Milan, Italy; antonello.rossetti@policlinico.mi.it; 5 Thoracic Surgery and Lung Transplant Unit, Fondazione IRCCS Ca’ Granda-Ospedale Maggiore Policlinico, 20122 Milan, Italy; ilaria.righi78@gmail.com (I.R.); davide.tosi@policlinico.mi.it (D.T.); 6 Respiratory Unit & Cystic Fibrosis Adult Center, Fondazione IRCCS Ca’ Granda-Ospedale Maggiore Policlinico, 20122 Milan, Italy; letizia.morlacchi@gmail.com; 7 Department of Anesthesia and Critical Care, Fondazione IRCCS Istituto Nazionale dei Tumori, 20133 Milan, Italy

**Keywords:** ex vivo lung perfusion, glycocalyx, coagulation, inflammasome, lung transplantation

## Abstract

The acceptable duration of donor warm ischemia time (DWIT) after cardiocirculatory death (DCD) is still debated. We analyzed the biomolecular profile and function during ex vivo lung perfusion (EVLP) of DCD lungs and their correlation with lung transplantation (LuTx) outcomes. Donor data, procurement times, recipient outcomes, and graft function up to 1 year after LuTx were collected. During EVLP, the parameters of graft function and metabolism, perfusate samples to quantify inflammation, glycocalyx breakdown products, coagulation, and endothelial activation markers were obtained. Data were compared to a cohort of extended-criteria donors after brain death (EC-DBD). Eight DBD and seven DCD grafts transplanted after EVLP were analyzed. DCD’s DWIT was 201 [188;247] minutes. Donors differed only regarding the duration of mechanical ventilation that was longer in the EC-DBD group. No difference was observed in lung graft function during EVLP. At reperfusion, “wash-out” of inflammatory cells and microthrombi was predominant in DCD grafts. Perfusate biomolecular profile demonstrated marked endothelial activation, characterized by the presence of inflammatory mediators and glycocalyx breakdown products both in DCD and EC-DBD grafts. Early graft function after LuTx was similar between DCD and EC-DBD. DCD lungs exposed to prolonged DWIT represent a potential resource for donation if properly preserved and evaluated.

## 1. Introduction

Lung transplantation (LuTx) is the only viable option for patients with end-stage lung diseases. Unfortunately, donor lung graft availability is limited, with only 30–50% [1] of multi-organ donors being suitable for lung donation. Thus, to expand the lung donor pool beyond donation after brain death (DBD), donation after cardiocirculatory death (DCD) has recently gained renewed attention [2]. Theoretically, DCD grafts are spared the cytokine and catecholamine storm associated with brain death and, uncontrolled DCD in particular, could be protected from ventilator-induced lung injury and be free from bacterial colonization/infection associated with mechanical ventilation (referred only to DCD-II). On the other hand, DCD lung grafts suffer from longer in situ donor warm ischemia time (DWIT) and, therefore, may have higher rates of primary graft dysfunction (PGD) [3] due to cellular bioenergetics failure [4]. Several protocols for organ retrieval following uncontrolled or controlled DCD have been developed [5], employing normothermic or hypothermic in situ preservation strategies. A wide range of DWIT has been reported without definitive data regarding rates of lung graft acceptability.

According to Italian law, 20 min of an isoelectric electrocardiogram (and thus at least 20 min of DWIT) are required to confirm cardiac death. As a result, a rapid retrieval technique is not possible. To thoroughly assess potentially eligible grafts and identify those suitable for LuTx in this context, our team developed a specific normothermic open lung [6] procurement procedure [7,8] combined with ex vivo lung perfusion (EVLP). In this setting, EVLP represents a unique platform to assess the pathophysiology of DWIT.

The aim of this study was to test whether lung grafts from DCD subjected to prolonged DWIT demonstrate a specific molecular signature potentially responsible for early graft injury. We used the EVLP platform to evaluate graft function and biomolecular markers of endothelial damage, glycocalyx breakdown products, inflammasome, and coagulation activation. These biomarkers were then correlated with early postoperative outcomes.

## 2. Materials and Methods

Our EVLP program has been previously described in detail elsewhere [9,10]. Notably, we employ an open atrium technique and Steen^TM^ solution (XVIVO Perfusion AB, Göteborg, Sweden) added to red blood cells (hematocrit 10–15%). The target perfusate flow is 40% of the donor’s estimated cardiac output and the duration of the procedure is typically 4 h. EVLP is employed in (1) lungs from DBD patients with a partial pressure of oxygen to fraction of inspired oxygen ratio (PaO_2_/FiO_2_) < 300 mmHg on a positive end-expiratory pressure of 5 cmH_2_O and/or with parenchymal consolidation on chest radiography or computed tomography after optimization of mechanical ventilation; (2) lungs from all donors undergoing veno-arterial extra-corporeal membrane oxygenation (ECMO) for cardio-circulatory support; (3) lungs from DCD-II to -IV [5,11]. Please see the Supplementary Materials for inclusion/exclusion criteria and procurement details.

### 2.1. Donors

Baseline demographic characteristics of the donors, including cause of death and lung function, were collected at the time of lung procurement. For extended-criteria (EC)-DBD, the Oto lung donor score [12] was calculated. In both groups, the total preservation time was calculated from pulmonary flush at the donor site to lung positioning into the recipient thorax and surgical WIT was calculated as the time from lung positioning into the recipient thorax to graft reperfusion. It is of note that DCD suffer from additional WIT between cardiac arrest and the start of graft preservation procedures (Table 1).

### 2.2. EVLP

Gas exchange was evaluated hourly by measuring the PaO_2_/FiO_2_ at an FiO_2_ of 100%, while deoxygenating venous perfusate by ventilating the membrane lung with nitrogen. Pulmonary vascular resistance (PVR), static lung compliance, and intrapulmonary shunt were computed according to standard formulae. Total dead space was measured by volumetric capnography at a single time point. Lung weight was recorded twice, once at the beginning and once at the end of the EVLP (please see the Supplementary Materials for EVLP procedure details).

#### Perfusate Biomolecular Profile

EVLP perfusate samples were collected at five time points: at the priming of the circuit (0), and after 60, 120, 180, and 240 min from the start of reperfusion. Standard biochemical tests were performed on whole perfusate while additional perfusate aliquots were collected into EDTA tubes and immediately centrifuged at 3000 rpm for 15 min at room temperature. Three mL of supernatant were stored at −80 °C for biomolecular analysis. A customized panel of mediators (Magnetic Luminex Screening Assay, R&D Systems INc, Minneapolis, MN, USA) was designed to explore the activation of different pathways potentially responsible for inflammation, glycocalyx shedding, chemotaxis, and endothelial function (Supplementary Materials Table S1). Fluorescence reading was carried out on a Luminex 200 analyzer (Luminex, Austin, CA, USA) and data were extracted with Luminex xPonent Software 4.2. Analytes, which resulted outside the calibration curve range defined for xMAP technology, were assessed by Enzyme-Linked Immunosorbent Assay (ELISA). Further analyses were performed to determine the concentration of total hyaluronan (HA) (R&D Systems) and of 8-hydroxy-2-deoxy Guanosine (8-OHdG) (DNA damage competitive ELISA kit, Thermo Fisher Scientific, Waltham, MA, USA). Absorbance was read between 450 and 570 nm on a multi-mode microplate reader (Synergy HTX, Biotek U.S, Winooski, VT, USA). Finally, the concentration of nitric oxide (NO) was evaluated through assessment of total NO metabolite (nitrites and nitrates) content (Sigma-Aldrich, St. Louis, MO, USA). All perfusate samples were run in duplicate. For all evaluated analytes, the concentration was corrected to account for the replacement of fresh Steen^TM^ solution that was performed hourly during EVLP (please see the formula applied in Supplementary Materials).

### 2.3. Recipients

Recipients’ demographic data, diagnosis, time spent on the transplant waiting list, lung mechanics, and gas exchange were collected. Lung allocation score (LAS) [13] was recorded at the time of the last evaluation preceding LuTx. Recipients’ incidence of multidrug-resistant pulmonary bacterial colonization, hospital admission at the time of LuTx, and requirement of home oxygen support or noninvasive mechanical ventilation or ECMO bridge to LuTx were recorded. The configuration and timing of intraoperative ECMO support, WIT duration, and quantity of blood products transfused during surgery were also collected. Upon admission to the intensive care unit (ICU), ventilator settings, respiratory mechanics, and gas exchange were recorded. PGD was assessed at 24 and 72 h post lung reperfusion [14] and patients receiving ECMO support were graded as PGD 3. The following outcomes were also recorded: requirement and duration of post-LuTx ECMO support, ICU length of stay, ventilator-free days at 28 days, incidence of reintubation and tracheostomy, renal and cardiac function, need for blood transfusions, and the fluid balance of the first 24 h post-Tx. Short-term post-Tx outcomes were evaluated at hospital discharge. Lung mechanics and gas exchange were recorded one year after LuTx.

### 2.4. Statistical Analysis

All continuous variables are presented as median [interquartile range]. Categorical variables are expressed as absolute number (percentage). Comparisons of continuous data were performed with Student’s t-test or the rank sum test, as appropriate, while the Chi-square test was used to compare categorical data. A two-way analysis of variance for repeated measures (ANOVA-RM) was performed for all EVLP parameters and perfusate biomolecular analytes [SigmaPlot 11.0 software]. Bonferroni’s correction was applied for repeated measures. To quantify between group differences, Cohen’s d effect size and odds ratio (OR) and the 95% confidence interval were calculated for continuous and categorical data, respectively. Pearson correlation was used to explore the relationship between the investigated biomolecular damage pathways and the duration of DWIT. An unsupervised agglomerative hierarchical cluster analysis was performed to identify any potential donor-specific signature or temporal trends in mediator release [NA-chip analyzer program https://sites.google.com/site/dchipsoft/home, accessed on 28 January 2021]. Within the DCD group, we tested the association between perfusate analyte concentration and total DWIT duration (ANOVA-RM) by dividing the total DWIT into two groups, above and below the median. Missing data accounted for <20% of the total data. A *p* value <0.05 was assumed to be statistically significant. Additional detail regarding the statistical analysis can be found in the online supplement.

## 3. Results

Fifteen successful EVLP procedures were performed at our institution from January 2018 to February 2020. Eight grafts were from EC-DBD and seven from DCD (5 DCD-II and 2 DCD-III).

### 3.1. Donor Characteristics

Demographic data for the donors are presented in Table 2. The total DWIT of the DCD cohort was partitioned into no-flow and low-flow time, which were 165 [161;190] and 62 [60;62] min for DCD-II and 136.5 [120;153] and 12 [11.5;12.5] min for DCD-III, respectively. The duration of cold ischemia preceding EVLP was similar between the DCD and EC-DBD group. None of the DCD donors had positive (>10^5^ bacterial CFU) bronchoalveolar lavage (BAL), whereas a positive BAL was detected in 50% of grafts obtained from EC-DBD donors (*p* = 0.077).

### 3.2. EVLP Parameters

**Figure 1:**
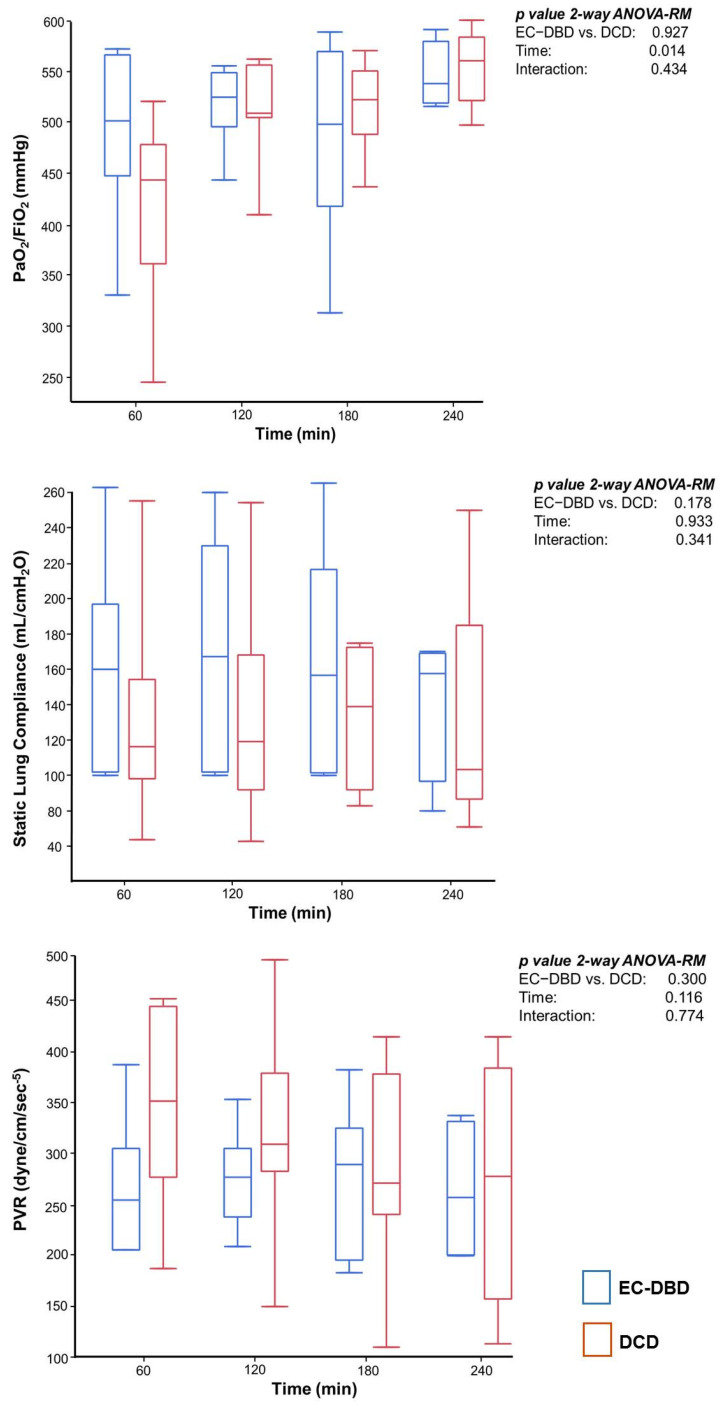
Lung Function. Lung function of EC-DBD (blue) and DCD (red) grafts along 4 h of EVLP. No differences were found between groups. As EVLP progressed, oxygenation progressively increased while minimal fluctuations in pulmonary vascular resistance and static compliance were observed in both groups. Abbreviations: PaO_2_/FiO_2_, partial pressure of oxygen to fraction of inspired oxygen ratio; PVR, pulmonary vascular resistance. Statistics: two-way ANOVA-RM, *p* value < 0.05 was assumed as statistically significant.

The duration of EVLP was similar between DCD and EC-DBD lungs (240 [200;300] vs. 190 [180;240] min; (*p* = 0.097; Cohen’s d = 0.682)). Figure 1 shows the lung function parameters recorded during EVLP. In both groups, oxygenation progressively increased during the procedure with a final PaO_2_/FiO_2_ of 561 [540;576] vs. 523 [502;557] mmHg in DCD and EC-DBD, respectively, corresponding to an intrapulmonary shunt of 31 [29;33] and 30 [24;34] % (*p* = 0.765; Cohen’s d = 0.167). Total dead space was 59 [56;60] vs. 56 [49;60] % (*p* = 0.710; Cohen’s d = 0.153). Static lung compliance and PVR remained stable throughout the procedure and were similar between groups. The increase in weight of the lung grafts was similar between groups (96 [71;315] vs. 66 [12;117] g DCD vs. EC-DBD, *p* = 0.215; Cohen’s d 0.712).

Perfusate metabolites, electrolytes, acid/base determinants, albumin, and hemo- and cytolysis marker concentrations are shown in Table 3.

**Figure 2:**
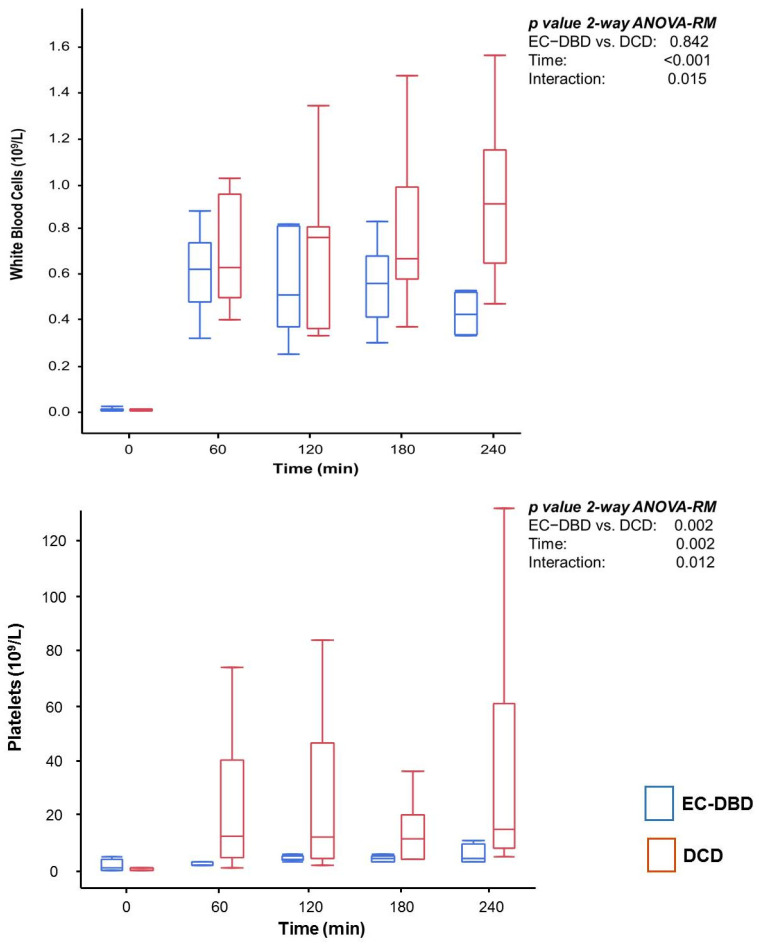
White Blood Cells and Platelets during EVLP. Appearance of white blood cells (WBC) and platelets in the EVLP perfusate of EC-DBD (blue) and DCD (red) grafts over 4 h of EVLP. No cellular blood components, other than erythrocytes, were present in the perfusate priming of the EVLP circuit. Similar levels of WBC appear immediately after graft reperfusion in both groups. Persistent WBC clearance occurs in the DCD grafts, but clearance decreases in the EC-DBD group. The “wash-out” of microthrombi in DCD graft leads to higher concentrations of platelets in the EVLP perfusate throughout the EVLP procedure. Statistics: two-way ANOVA-RM, *p* value < 0.05 was assumed as statistically significant.

The concentration of ALT (*p* = 0.037) increased in the DCD group, while there was no difference in Lactate Dehydrogenase levels (*p* = 0.375). Perfusate flow cytometry during EVLP is depicted in Figure 2.

### 3.3. Perfusate Biomolecular Profile

In the perfusate samples, at all time points, all molecules were detected, with the exception of VCAM-1. Notably, RANTES, IL-6, IL-8, PLA2G7, sRAGE, and HMGB-1 were abundantly released in the perfusate, whereas concentrations of IL-10, IL-1β, and TNF-α were low. VCAM-1 and nitrite were undetectable at all time points.

#### 3.3.1. Perfusate Differences between Donor Categories

Differences in perfusate factors across donor categories were investigated by means of ANOVA-RM. All the evaluated mediators showed increased concentrations during the EVLP procedure in both DCD and EC-DBD grafts (Supplementary Materials Figures S1–S5). The concentrations of endothelial glycocalyx breakdown products and matrix remodeling index TIMP-1 (Supplementary Materials Figure S1), as well as those of endothelial damage markers, and NO metabolites (Supplementary Materials Figure S2) did not differ between groups (all *p* > 0.05). The oxidative stress index, 8-OHdG (Supplementary Materials Figure S2), tended to be higher, but not significantly, in DCD perfusate compared to the EC-DBD group. Regarding markers involved in coagulation activation (Supplementary Materials Figure S3), a greater concentration of platelets (*p* = 0.002), PAI-1 (*p* = 0.060), and D-dimer (*p* = 0.056) was observed in the DCD lung perfusate samples, while the tissue factor level was significantly higher in the EC-DBD grafts (*p* = 0.003). Finally, although the release of inflammatory mediators was similar between groups (all *p* > 0.05, Supplementary Materials Figures S4 and S5), a trend towards higher Procalcitonin and Pentraxin-3 (Supplementary Materials Figure S4) concentrations was found in the EC-DBD group.

#### 3.3.2. Association between Perfusate Biomolecular Profile and DWIT

We investigated potential correlations between perfusate biomolecular profiles and total duration of DWIT in DCD grafts. Significant associations were identified between DWIT and the concentration of the following molecules: PAI-1 (R^2^ = 0.6225, *p* = 0.035), TIMP-1 (R^2^ = 0.7919, *p* = 0.008), HMGB1 (R^2^ = 0.7065, *p* = 0.018), and PLA2G7 (R^2^ = 0.7241, *p* = 0.015) (Supplementary Materials Table S3).

#### 3.3.3. Hierarchical Clustering Analysis

**Figure 3:**
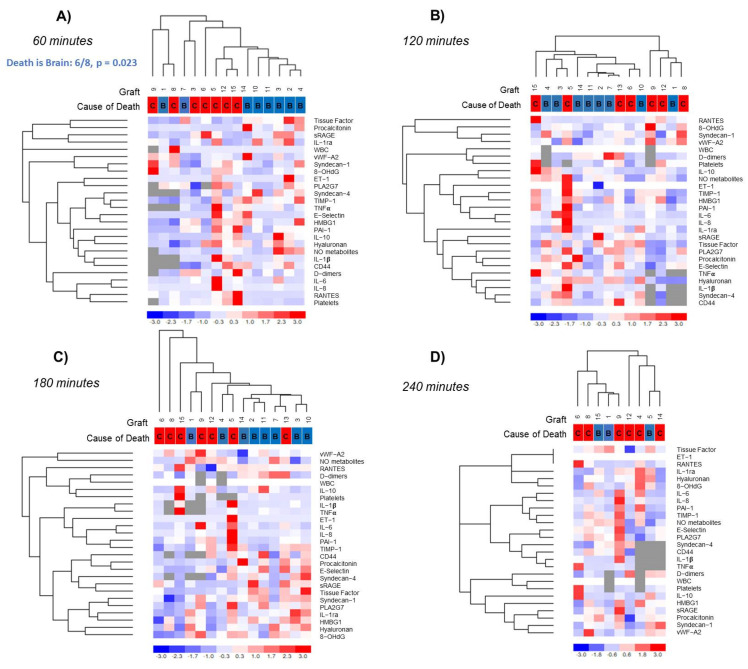
Hierarchical cluster analysis of analyte perfusate concentration. Agglomerative hierarchical clustering was performed to investigate whether a donor-specific signature could be identified in the molecular profile of the perfusate. Independent analyses were carried out at (**A**) 60, (**B**) 120, (**C**) 180, and (**D**) 240 min. Unsupervised analysis revealed a donor-specific pattern at 60 min, with six out of eight EC-DBD patients grouped in a single cluster (*p* = 0.0230). This distinctive profile was not recognizable at the subsequent time points. Cause of death was referred to as B for EC-DBD (blue) and C for DCD (red) donors. Cluster analysis was performed using dCHIP software (clustering method: average linkage; distance metric: 1—Spearman’s rank correlation). Columns identify cases, while rows denote the parameters evaluated in perfusate. The degree of color saturation reflects the magnitude of the mediator concentration, as indicated in the color scale.

Unsupervised clustering analysis was performed to investigate the overall kinetic of mediator release in the perfusate during EVLP and to assess whether donor-specific signatures were recognizable. The analysis identified a distinctive perfusate profile between the first 60 min of reperfusion, absent in subsequent time points (*p* = 0.0004, Supplementary Materials Figure S6). In addition, the following clusters of variables with similar profiles across samples were observed (data not shown): (1) CCL5/RANTES, D-dimers, platelets; (2) Syndecan-1, vWF-A2, 8-OHdG; (3) tissue factor, Procalcitonin, RAGE, HA; (4) Endothelin-1, TIMP-1, NO metabolites, PAI-1. Based on the results of this preliminary evaluation, additional unsupervised cluster analysis was independently performed at each time interval (Figure 3). The results of the first analysis were confirmed: a donor-specific signature was identified at 60 min of EVLP (*p* = 0.023, Figure 3A), while no donor-dependent biomolecular profile was observed at subsequent time points (Figure 3B–D).

### 3.4. Recipient Characteristics and Outcomes

Table 4 shows the recipient characteristics at the time of LuTx.

The median LAS score of our patients was 40 (39–49) and 40 (37–44) for EC-DBD and DCD, respectively, thus indicating an intermediate disease severity, which could be due to the high incidence of cystic fibrosis in the study groups.

Bilateral LuTx was performed in all cases and no ECMO as a bridge to LuTx was necessary. Although no statistically significant difference was observed between patient cohorts, there was a trend towards a higher proportion of EC-DBD recipients requiring intraoperative veno-arterial ECMO compared to DCD recipients, with a subsequent higher volume of blood products transfused. The most common indication for intraoperative veno-arterial ECMO was inability to tolerate one lung ventilation during pneumonectomy of the first lung.

Recipients’ post-LuTx outcomes and early pulmonary function are summarized in Table 5.

We did not observe any difference in gas exchange and lung mechanics at ICU admission (all *p* > 0.05). Short-term (maximum 3 days) postoperative veno-venous ECMO support was required in two EC-DBD and one DCD patients, and no patient received a tracheostomy. The incidence of PGD 3 was 37 vs. 28% (*p* = 0.999; OR 1.5 [13.2;0.2]) at 24 h and 12 vs. 14% (*p* = 0.999; OR 0.9 [0.1;16.8]) at 72 h in the EC-DBD and DCD groups, respectively. Five patients from each group required short-term inotropic support. No major bleeding occurred during the ICU stay and acute kidney injury incidence was similar between groups. Although the length of hospital stay was slightly shorter in the DCD group, recovery of lung mechanics and gas exchange was achieved in both groups with optimal oxygenation both at rest and during the six-minute walking test. Twelve months after Tx, 25% and 14% of patients, in the EC-DBD and DCD group, respectively, had acute rejection. Unfortunately, one recipient in the DCD group died 25 days after LuTx due to acute antibody-mediated rejection unresponsive to plasmapheresis [15]. In the EC-DBD group, two patients died within the first year after LuTx, both as consequence of post-Tx lymphoproliferative disease with cerebral and gastrointestinal involvement. Survival was 75% in the EC-DBD group and 86% in the DCD group one year after LuTx and these rates remain valid at the time of writing.

## 4. Discussion

Acceptable DWIT for lung donation in the setting of DCD has been reported as up to 90 min in Australia [16], 120 min in the UK [17], and a maximum of 150 min in Spain [18]. Several previous case series described an average total DWIT (considering both DCD-II and -III) between 20–40 min [19]. However, a recent retrospective analysis of a large DCD cohort did not find a correlation between DWIT and early and late post-LuTx outcomes [20,21]. In Italy, DCD lung donation represents a challenge due to legislative constraints as 20 min of asystole are required to confirm cardiocirculatory death, making rapid retrieval strategies unfeasible. In 2015, the Milan Lung Transplant Center, with the collaboration of the local organ procurement organization, Nord Italia Transplant, set up a program to perform a nonrapid and normothermic lung procurement from DCD-II patients and, subsequently, DCD-III [7,8] patients. By keeping the graft inflated during the ischemic phase, tissue oxygen delivery is guaranteed and organ viability can be preserved. Organ function is ultimately assessed during 4 h of EVLP. In the present case series, the median duration of total DWIT among DCD was longer than 3 h. In this prospective exploratory study, we exploited the extremely controlled reperfusion platform that is used during EVLP to evaluate whether DCD lungs exposed to prolonged DWIT show a specific pathophysiological signature compared to grafts from EC-DBD.

We observed a significant release of inflammatory mediators and glycocalyx/endothelium damage markers in both DCD and EC-DBD grafts, with higher levels of white blood cells, platelets, and coagulation factor in the DCD cohort. Cluster analysis indicated that the most significant differences in the perfusate biomolecular profiles between donor categories can be observed within the first hour of ex vivo perfusion. Lungs from DCD donors undergoing prolonged DWIT and EC-DBD were qualitatively comparable in terms of both pulmonary function at the end of EVLP and immediate and short-term clinical outcomes.

We did not observe significant clinical differences between the two donor cohorts, except for mechanical ventilation duration before procurement. No clinically meaningful difference in recipient characteristics was observed, possibly due to the use of LAS-based graft assignment. At the end of the EVLP procedure, both treatment groups had good oxygenation for LuTx, following a progressive increase in PaO_2_/FiO_2_ during the procedure. In both groups we measured similarly elevated dead space fraction despite normal PVR, possibly due to the relatively low perfusate flow applied during EVLP. Static lung compliance was stable and within the normal range in both DCD and EC-DBD cohorts.

Several preclinical and clinical studies have attempted to define the biomolecular profile of lung grafts during EVLP by analyzing the circulating perfusate and, when possible, the pulmonary tissue [22,23,24,25,26,27,28,29]. In this study, where the DCD cohort consisted of mainly uncontrolled donors (five out of seven), we observed a high number of neutrophils and lymphocytes in the EVLP perfusate of both groups, immediately after reperfusion. Interestingly, the white blood cell count stabilized in the EC-DBD grafts, while in the DCD group it progressively rose, suggesting the persistence of a clearance effect. Despite the high dose of steroids administered in the EVLP perfusate to prevent ischemia-reperfusion (I/R) damage, we observed elevated proinflammatory mediators both in EC-DBD and DCD grafts. Elevated levels of IL-6 and IL-8 in recipient plasma post-reperfusion has been associated with the incidence of PGD in several studies [30]. Overall, our study found no association between inflammatory cytokine production during EVLP and donor type. In fact, although DCD grafts presented both higher levels of inflammatory mediator, even if not statistically significant, and wider distribution ranges than the EC-DBD lungs, we did not record a higher incidence of PGD in the DCD group. Similar to previous reports, we observed low levels of IL-10 [23], IL-8, and IL-1β in all but two DCD cases [31].

DCD donors showed higher concentrations of oxidative stress (i.e., 8-OHdG) and cytolysis markers (i.e., ALT and LDH), possibly due to prolonged DWIT. With the exception of ALT concentrations, however, 8-OHdG and LDH levels in the DCD lungs were not significantly higher. DCD lungs are at high risk of deterioration due to DWIT between cardiac arrest and organ procurement. Additionally, free radicals that form during reoxygenation contribute to tissue injury. For this reason, our recovered DCD lungs after a long period of DWIT followed by cold ischemia and 4 h of EVLP should have shown significant cell damage, evidenced by a massive release of LDH. In contrast, in our samples, the LDH release, a strong indicator of oxidant-mediated cell damage, was only partially, but not significantly, greater in DCD lung perfusate than in EC-DBD, in agreement with the levels of oxidative stress markers analyzed.

Contrary to previous studies [32], all but one DCD graft showed low ET-1 levels (i.e., a marker of endothelial dysfunction) with preserved NO production throughout the EVLP procedure, while endothelial adhesion molecule concentration increased starting at 120 min in both groups. Similarly to Sladden et al. [33], we observed an increase in glycocalyx degradation in both cohorts, suggesting that glycocalyx integrity could be a potential target for graft protection from I/R injury [34,35]. The DCD group had higher platelets, D-dimer, and PAI-1 with enhanced tissue factor consumption, possibly due to vascular stasis and microvascular thrombi formation during the DWIT phase. In the DCD setting, EVLP could guarantee microthrombi clearance before LuTx. Positive correlations between prolonged DWIT duration and TIMP-1, PAI-1, HMGB1, and PLA2G7 were found among the DCD grafts, indicating that with prolongation of DWIT there was an increase in the remodeling of the extracellular matrix composition, an increase in microthrombosis, and an intensification of inflammation related to necrosis and macrophages/T lymphocytes cells activation, respectively.

Overall, because lung function and weight were similar between the two groups, the slightly more pronounced inflammation, oxidative stress, and procoagulatory activation in DCD lungs was not associated with clinically relevant alterations in graft properties and may only represent a “wash-out” effect. Furthermore, we cannot confirm the paradigm of a more pronounced inflammatory response associated with brain death-induced catecholamine storm [24] in DBD donor grafts. On the other hand, we found a trend towards higher Procalcitonin and Pentraxin-3 levels in EC-DBD lungs, probably due to airway bacterial colonization/infections of DBD donors who, contrary to DCD-II donors (prevalent in our DCD cohort), undergo several days of mechanical ventilation prior to procurement and may be exposed to brain-death related immunodepression. Compared to a larger population of LuTx recipients at our institution [10], the current cohort of DCD exposed to prolonged DWIT, despite being small, showed no differences in both respiratory and nonrespiratory related outcomes assessed during the ICU stay, hospital stay, and 1-year after LuTx.

This study has several limitations. First, the small sample size can lead to underestimation of the biological and functional differences between the studied cohorts. Studies with more patients are necessary to confirm our observations. However, grafts from DCD-II and DCD-III undergoing prolonged DWIT represent an “extreme” setting and we hypothesized that these patients could have exhibited a specific pattern of lung injury, if present. Second, in the analysis, we did not include either standard DBD donors, as these grafts are not suitable candidates for EVLP, nor lungs deemed unsuitable for Tx at the end of procedure, as they were confusing and not comparable with other lungs due to parenchymal bleeding, massive edema, and airways plasma-leak, which made it impossible to continue the EVLP technique beyond one hour. Lastly, biomolecular analyses were performed exclusively by measuring soluble mediators without the support of tissue gene expression analyses and histological examination. Although it is plausible that RNA expression and other metrics could provide further insights into biological differences between the groups studied, we preferred to avoid tissue sampling to preserve the lung parenchyma before LuTx. Furthermore, the biomolecular evaluation of the EVLP perfusate at multiple time points easily provides useful information on the kinetic and the magnitude of the lung stress response over the EVLP procedure.

## 5. Conclusions

Compared to EC-DBD, lungs from DCD exposed to prolonged DWIT are qualitatively comparable in pulmonary function, inflammasome activation, endothelial damage, and glycocalyx shedding. EVLP may promote microthrombi clearance in DCD grafts. Our results support the acceptability of lung grafts from DCD donors with prolonged DWIT after EVLP evaluation.

## Figures and Tables

**Table 1:** DCD warm ischemia time.

DCD-II	No Flow (min)	Low Flow (min)	Total DWIT (min)
DCD n° 1	5 + 185 = 190	115	305
DCD n° 3	14 + 125 = 139	62	201
DCD n° 4	6 + 155 = 161	26	187
DCD n° 5	9 + 199 = 208	62	270
DCD n° 7	10 + 155 = 165	60	225
**DCD-III**	**Low Flow (min)**	**No Flow (min)**	**Total DWIT (min)**
DCD n° 2	11	103	140
DCD n° 6	13	170	190

For DCD-II, total DWIT is partitioned into (1) *No-flow time*: from cardiac arrest to the start of cardiopulmonary resuscitation added to (+) the time from the initiation of the no-touch period until the lung cold flush is performed during graft procurement and (2) *Low-flow time*: from the beginning of cardiopulmonary resuscitation until the interruption of resuscitation maneuvers. For DCD-III, after the withdrawal of life sustaining therapies, DWIT is partitioned into (1) *Low-flow time:* from the time the patient reaches a systolic arterial pressure below 50 mmHg until cardiac arrest occurs and (2) *No-flow time*: from cardiac arrest until the initiation of cold flush of the lung. Abbreviations: DCD, donors after cardiocirculatory death; DWIT, donor warm ischemia time.

**Table 2:** Donor characteristics.

	EC-DBD (*n* = 8)	DCD (*n* = 7)	*p*-Value	Effect Size
**Age, years**	37.7 [26.5;46]	54 [46.5;56]	0.147	0.80
**Male Sex, *n* (%)**	7 (87)	6 (86)	1	1.2 [0.1;22.9]
**BMI, kg/m^2^**	27.0 [24.3;31]	27.7 [25.3;28.7]	0.672	0.25
**Cause of death, *n* (%)**			0.016	
**DBD**				
**Cerebrovascular**	1 (13)	-		
**Trauma**	5 (62)	-		
**Post-anoxic**	0 (0)	-		
**Other**	2 (25)	-		
**DCD**				
**Class II**	-	5 (71)		
**Class III**	-	2 (29)		
**Total in-situ WIT, min**	-	201 [185;247]		
**MV Duration, days**	2 [2;4]	0 [0;0.8]	0.009	1.250
PaO_2_/FiO_2_, mmHg *	304 [245;339]	-		
OTO score *	8 [6;10]	-		
**CIT pre-EVLP**	290 [170;290]	200 [180;210]	0.165	1.000

Among DCD donors, lung gas exchange was not recorded due to the measurements being not feasible (DCD-II) or unreliable (DCD-III) due to low patient cardiac output or the presence of extracorporeal support. * OTO score and PaO_2_/FiO_2_ are considered only in DBD donors. Abbreviations: BMI, body mass index; CIT pre-EVLP, cold ischemia time before ex vivo lung perfusion; DCD, donors after cardiocirculatory death; EC-DBD, extended-criteria donors after brain death; MV, mechanical ventilation; PaO_2_/FiO_2_, arterial partial pressure of Oxygen to fraction of inspired Oxygen ratio; WIT, warm ischemia time.

**Table 3:** EVLP Perfusate Composition.

	Group	0	60	120	180	240	*p* Value Group	*p* Value Time	*p* Value Interaction
**Glucose (mg/dL)**	EC-DBD	239 ± 5	184 ± 5	158 ± 6	137 ± 5	108 ± 7	0.110	<0.001	0.874
	DCD	252 ± 5	198 ± 5	176 ± 6	148 ± 6	131 ± 6			
**Lactate (mmol/L)**	EC-DBD	2.4 ± 0.3	6.2 ± 0.3	9.2 ± 0.4	11.2 ± 0.3	14.2 ± 0.5	0.638	<0.001	0.279
	DCD	2.1 ± 0.4	6.8 ± 0.3	9.8 ± 0.4	12.3 ± 0.4	14.0 ± 0.4			
**Albumin (g/dL)**	EC-DBD	6.0 ± 0.1	5.9 ± 0.1	5.7 ± 0.1	5.8 ± 0.1	5.5 ± 0.2	0.626	0.117	0.375
	DCD	5.7 ± 0.2	5.7 ± 0.2	5.6 ± 0.2	5.6 ± 0.2	5.7 ± 0.2			
**pH**	EC-DBD	7.08 ± 0.02	7.00 ± 0.01	6.95 ± 0.01	6.96 ± 0.01	6.88 ± 0.02	0.489	<0.003	0.006
	DCD	7.09 ±0.04	6.98 ± 0.01	6.96 ± 0.01	6.96 ± 0.01	6.98 ± 0.01			
**pCO_2_ (mmHg)**	EC-DBD	38 ± 1	32 ± 1	31 ± 1	30 ± 1	27 ± 1	0.762	<0.001	0.849
	DCD	34 ± 1	33 ± 1	31 ± 1	30 ± 1	27 ± 1			
**HCO_3_^−^ (mmol/L)**	EC-DBD	11.5 ± 0.4	8.7 ± 0.3	7.7 ± 0.3	7.0 ± 0.3	5.3 ± 0.4	0.996	<0.001	0.109
	DCD	11.0 ± 0.5	8.2 ± 0.3	7.0 ± 0.3	6.8 ± 0.3	6.3 ± 0.3			
**Na^+^ (mmol/L)**	EC-DBD	147 ± 1	153 ± 0	156 ± 1	159 ± 1	165 ± 1	0.012	<0.001	0.107
	DCD	146 ± 1	150 ± 1	154 ± 1	157 ± 1	159 ± 1			
**K^+^ (mmol/L)**	EC-DBD	7.5 ± 0.1	6.6 ± 0.1	6.4 ± 0.1	6.2 ± 0.1	6.3 ± 0.1	0.874	<0.001	0.905
	DCD	7.6 ± 0.2	6.7 ± 0.1	6.5 ± 0.1	6.3 ± 0.1	6.3 ± 0.1			
**Ca^2+^ (mmol/L)**	EC-DBD	0.74 ± 0.01	0.79 ± 0.01	0.81 ± 0.01	0.83 ± 0.01	0.85 ± 0.01	0.040	<0.001	0.009
	DCD	0.75 ± 0.01	0.83 ± 0.01	0.86 ± 0.01	0.86 ± 0.01	0.86 ± 0.01			
**Free Hb (mg/dL)**	EC-DBD	3.5 ± 1.4	7.6 ± 1.3	10.0 ± 1.3	11.0 ± 1.3	18 ± 2.0	0.616	<0.001	0.145
	DCD	3.5 ± 1.8	10.0 ± 1.5	9.8 ± 1.4	11.8 ± 1.5	12 ± 1.5			
**ALT (U/L)**	EC-DBD	1 ± 1	3 ± 1	5 ± 1	6 ± 1	6 ± 2	0.037	<0.001	<0.001
	DCD	4 ± 2	17 ± 2	21 ± 2	23 ± 2	24 ± 2			
**CPK (U/L)**	EC-DBD	1 ± 65	219 ± 65	233 ± 72	259 ± 72	455 ± 92	0.136	<0.001	0.147
	DCD	0 ± 91	293 ± 77	483 ± 91	629 ± 89	716 ± 91			
**LDH (U/L)**	EC-DBD	10 ± 10	95 ± 10	135 ± 11	163 ± 11	210 ± 16	0.375	<0.001	0.127
	DCD	15 ± 20	137 ± 20	191 ± 20	226 ± 23	255 ± 24			

Abbreviations: ALT, Aspartate Aminotransferase; Ca^2+^, ionized Calcium; CPK, Creatine Phosphokinase; DCD, donors after cardiocirculatory death; EC-DBD, extended-criteria donors after brain death; Free Hb, perfusate free hemoglobin; HCO^3−^, Bicarbonate; LDH, Lactate Dehydrogenase; Na^+^, Sodium; pCO_2_, partial pressure of Carbon Dioxide; K^+^, Potassium.

**Table 4:** Recipient characteristics.

	EC-DBD (*n* = 8)	DCD (*n* = 7)	*p* Value	Effect Size
***Pre-operative***				
**Age, years**	35 [24;41]	32 [28;48]	0.683	0.207
**Male Sex, *n* (%)**	7 (88)	5 (71)	0.569	2.8 [0.2;40.1]
**BMI, kg/m^2^**	21.3 [17.0;27.1]	21.1 [18.0;22.2]	0.867	0.230
**Time on WL, days**	172 [103;237]	356 [289;446]	0.069	1.026
**Disease, *n* (%)**			0.506	
**Cystic Fibrosis**	6 (76)	5 (71)		
**Pulmonary Fibrosis**	1 (12)	0 (0)		
**COPD**	1 (12)	2 (29)		
**LAS**	40 [39;49]	40 [37;44]	0.237	0.679
**PaO_2_/FiO_2_, mmHg**	278 [235;288]	258 [246;282]	0.200	0.358
**PaCO_2_, mmHg**	44 [40;50]	44 [44;51]	0.866	0.111
**FVC, %**	65 [44;74]	38 [34;43]	0.043	1.269
**FEV_1_, %**	39 [24;73]	24 [16;25]	0.059	1.360
**Colonized, *n* (%)**	6 (75)	5 (71)	0.999	1.2 [0.1;11.9]
**Hospitalized, *n* (%)**	2 (25)	1 (17)	0.999	2.0 [0.1;28.4]
**LV ejection fraction,%**	57 [54;61]	60 [59;61]	0.452	0.372
**NIMV dependent, *n* (%)**	5 (63)	6 (88)	0.569	0.3 [0.0;3.6]
**O_2_ dependent, *n* (%)**	7 (88)	7 (100)	0.999	-
***Intra-operative***				
**Duration of Surgery, min**	592 [524;693]	594 [460;625]	0.521	0.340
**Total CIT 1st lung, min**	604 [490;655]	565 [524;636]	0.974	0.231
**Total CIT 2nd lung, min**	810 [670;896]	780 [719;879]	0.960	0.057
**WIT 1st lung, min**	79 [77;88]	79 [72;97]	0.692	0.182
**WIT 2nd lung, min**	69 [65;83]	64 [57;85]	0.591	0.282
**ECMO, *n* (%)**	6 (75)	3 (43)	0.315	4.0 [0.4;35.8]
**VA-ECMO, *n***	6	3		
**ECMO 1st lung, *n***	4	2		
**ECMO 2nd lung, *n***	2	1		
**Transfusions, units**				
**PRBC**	7 [5;10]	2 [1;5]	0.020	1.432
**FFP**	3 [0;11]	2 [1;5]	0.213	0.703
**PLT**	0 [0;6]	0 [0;0]	0.383	0.770

Abbreviations: BMI, body mass index; CIT, cold ischemia time; COPD, chronic obstructive pulmonary disease; DCD, donors after cardiocirculatory death; EC-DBD, extended-criteria donors after brain death; FEV_1_, forced expiratory volume within the 1st second; FFP, fresh frozen plasma; FVC, forced vital capacity; LAS, lung allocation score; LV, left ventricle; NIMV, noninvasive mechanical ventilation; PaCO_2_, arterial partial pressure of Carbon Dioxide; PaO_2_/FiO_2_, arterial partial pressure of Oxygen to fraction of inspired Oxygen ratio; PRBC, packed red blood cells; PLT, platelets; Time on WL, time on waiting list; VA-ECMO, veno-arterial extracorporeal membrane oxygenation; WIT, warm ischemia time.

**Table 5:** Recipient outcomes.

	EC-DBD (*n* = 8)	DCD (*n* = 7)	*p* Value	Effect Size
***ICU Admission***				
**Intubated, *n* (%)**	8 (100)	7 (100)	0.999	-
PaO_2_/FiO_2_, mmHg *	242 [168;333]	228 [183;260]	0.638	0.270
**PEEP, cmH_2_O**	10 [10;13]	11 [10;12]	0.694	0.165
**Compliance, ml/cmH_2_O**	42 [37;43]	37 [30;57]	0.955	0.309
**Shunt, %**	11 [7;18]	14 [10;17]	0.777	0.151
***ICU Discharge***				
**28-days ventilator free, days**	27 [22;28]	26 [25;28]	0.779	0.261
Post-operative ECMO, *n* (%) **	2 (25)	1 (14)	0.999	1.7 [0.1;24.3]
**Tracheostomy, *n* (%)**	0 (0)	0 (0)	-	-
**ICU LOS, days**	3 [1;8.5]	3 [2;10]	0.613	0.165
**ICU readmission, *n* (%)**	0 (0)	1 (14)	0.467	-
**ICU survival, *n* (%)**	8 (100)	6 (86)	0.467	-
**24h post-LuTx fluid balance, mL**	621 [62;1300]	−259 [−505;523]	0.525	0.339
**Inotropic support, *n* (%)**	5 (63)	5 (71)	1	0.7 [0.1;5.9]
**Post-operative transfusions, units**				
**PRBC**	0 [0;2]	1 [0;2]	0.535	0.000
**FFP**	0 [0;0]	0 [0;0]	0.999	0.534
**PLT**	0 [0;0]	0 [0;0]	0.710	0.534
Perioperative AKI ***			0.368	-
**Stage 1**	3	3		
**Stage 2**	0	1		
**Stage 3**	1	0		
**PGD 3 at 24 h, %**	37	28	0.999	1.5 [13.2;0.2]
**PGD 3 at 72 h, %**	12	14	0.999	0.9 [0.1;16.8]
***Hospital Discharge***				
**Hospital LOS**	27 [22;35]	20 [17;25]	0.035	0.932
**Post-Tx Hospital Mortality, *n* (%)**	2 (25)	1 (14)	1	2.0 [28.3;0.1]
**Resting SpO_2_, %**	98 [97;98]	97 [96;98]	0.229	0.000
**FEV_1_, %**	60 [51;74]	57 [37;65]	0.295	0.674
**FVC, %**	54 [48;65]	51 [39;58]	0.466	0.422
**6MWT, m**	370 [309;482]	400 [335;459]	0.846	1.689
**Mean 6MWT SpO_2_, %**	96 [95;97]	96 [95;97]	0.782	0.000
***12-months post LuTx***				
**Survival, *n* (%)**	6 (75)	6 (86)	1	0.5 [0.1;7.1]
**Acute R** **ejection Index, *n* (%)**	2 (25)	1 (14)	1	2 [28.4;0.1]
**Resting SpO_2_, %**	99 [99;100]	99 [99;100]	0.563	0.345
**FEV_1_, %**	88 [79;94]	79 [64;94]	0.682	0.244
**FVC, %**	83 [76;89]	82 [69;95]	0.886	0.085

* PaO_2_/FiO_2_ in patients undergoing post-operative ECMO is not considered. ** It is intended as a continuation of intraoperative ECMO. Duration, 2.5 days for EC-DBD and 3 days for DCD. *** AKI staging has been calculated according to the KDIGO classification. Abbreviations: AKI, acute kidney injury; DCD, donors after cardiocirculatory death; EC-DBD, extended-criteria donors after brain death; ECMO, extracorporeal membrane oxygenation; FEV1, forced expiratory volume within the 1st second; FFP, fresh frozen plasma; FVC, forced vital capacity; ICU, intensive care unit; LOS, length of stay; LuTx, lung transplantation; PaO_2_/FiO_2_, arterial partial pressure of Oxygen to fraction of inspired Oxygen ratio; PEEP, positive end-expiratory pressure; PGD, primary graft dysfunction; PLT, platelets; PRBC, packed red blood cells; 6MWT, six-minute walking test; SpO_2_, peripheral hemoglobin saturation of Oxygen.

## Supplementary Materials

The following supporting information can be downloaded at: https://www.mdpi.com/article/10.3390/jcm11113066/s1, Table S1: Biomolecular Analysis: pathways and techniques. Table S2: Biomolecular Analysis Assay. Table S3: Pearson Correlation analysis between donor warm ischemia time and biomolecular profile. Figure S1: Glycocalyx Shedding and tissue remodeling indices. Figure S2: Endothelium Damage. Figure S3: Coagulation Activation. Figure S4: Innate Immunity Activation. Figure S5: Inflammatory Mediators. Figure S6: Unsupervised Cluster Analysis.

## Abbreviations

EC-DBD	Extended-criteria donors after brain death
DCD	Donors after cardiocirculatory death
DWIT	Donor warm ischemia time
EVLP	Ex vivo lung perfusion
ICU	Intensive care unit
LuTx	Lung transplantation
NO	Nitric Oxide
PaO_2_/FiO_2_	Partial pressure of Oxygen to fraction of inspired Oxygen ratio
PGD	Primary graft dysfunction
PVR	Pulmonary vascular resistance
