# Supplementary material for: Lung Biomolecular Profile and Function of Grafts from Donors after Cardiocirculatory Death with Prolonged Donor Warm Ischemia Time
Source: J Clin Med. 2022 May 29;11(11):3066. doi: 10.3390/jcm11113066 (PMC9181171; doi:10.3390/jcm11113066)
Supplement: Supplementary file 1 [file jcm-11-03066-s001.zip › jcm-1670470-supplementary.pdf]

Lung Biomolecular Profile and Function of Grafts from Donors after Cardiocirculatory Death with Prolonged Donor Warm Ischemia Time

Francesca Gori; Jacopo Fumagalli; Caterina Lonati; Andrea Carlin; Patrizia Leonardi; Osvaldo Biancolilli; Antonello Rossetti; Ilaria Righi; Davide Tosi; Alessandro Palleschi; Lorenzo Rosso; Letizia Corinna Morlacchi; Francesco Blasi; Luigi Vivona; Gaetano Florio; Vittorio Scaravilli; Franco Valenza; Alberto Zanella and Giacomo Grasselli.

Supplementary Materials

Supplement Methods

EVLP program inclusion criteria:

Extended-Criteria DBD:

- age >55 years
- PaO_2/FiO_2 <300mmHg on PEEP 5 cmH_2O and FiO_2 100% after lung recruitment
- parenchymal consolidation present on chest radiograph or computed tomography
- donors on Veno-Arterial ECMO
- brain death occurring after hanging injury

DCD class II:

- no-flow time < 15 minutes

- low-flow time < 60 minutes

- not a candidate for extracorporeal cardiopulmonary resuscitation

DCD class III:

- low-flow time < 50 minutes

EVLP program exclusion criteria:

- age > 65 years
- smoking history > 20 pack/year
- massive lung contusion
- inhalation/aspiration injury
- pneumonia
- sepsis
- malignancy
- in all DCD classes total WIT should not exceed 240 minutes

Lung procurement:

After preparing the organ for harvesting and cannulating the pulmonary artery, 500 mcg of prostaglandin E1 was injected into the pulmonary artery, the aorta and caval veins cross clamped, the left ventricular apex or ascending aorta were transected and, finally, anterograde pulmonary flush was performed with 60 ml/kg of cold (4-8 °C) Perfadex™. Retrograde pulmonary flush was performed at the time of the back-table by using 250 ml of Perfadex™ for each pulmonary vein.

EVLP procedure:

Broad spectrum antibiotics, heparin and methylprednisolone were added to the perfusate. The circulating perfusate was replaced with a known volume of clear Steen™ solution every hour: specifically, 500 mL after the 1st hour and 250 mL at the 2nd and 3rd hour. The procedure included the first hour of graft warming followed by 3 hours of steady state. Perfusate flow, temperature, pulmonary artery pressure (PAP), tidal volume, plateau airway pressure and Positive End-Expiratory Pressure (PEEP) were recorded every hour to calculate pulmonary vascular resistance (PVR) and static lung compliance. To evaluate gas exchange, the membrane lung was ventilated every hour with nitrogen to de-oxygenate the perfusate and PaO_2/FiO_2 was measured from the samples drawn from the pulmonary veins while ventilating the graft at an FiO_2 of 100% to exclude venous admixture.

Perfusate Analysis:

Electrolytes (K^+, Na^{2+}, Cl^-, Ca^2) and metabolites (glucose and lactate) were measured by a blood gas analyzer (ABL 800Flex; Radiometer Medical ApS, Brønshøj, Denmark). Standard blood tests were performed on whole perfusate: platelets and white blood cell count, hemoglobin and albumin concentrations were tested. Alanine aminotransferase (ALT), creatin phosphokinase (CPK), and lactate dehydrogenase (LDH), a non-specific marker of cytolysis, and serum free hemoglobin, a marker of hemolysis, were also measured. Blood urea nitrogen and ammonium were measured as indices of protein cell catabolism.

To quantify the burden of glycocalyx shedding, we measured the concentration of total hyaluronan and its receptor CD44, Syndecan-1, and Syndecan-4 in the perfusate. Furthermore, tissue inhibitor of metalloproteinase 1 (TIMP-1) was tested as an indirect measure of the activity of metalloproteinase-1, -7, and -9. Endothelial damage was evaluated by dosing Endothelial-Selectin (E-Selectin), Vascular Cell Adhesion Molecule 1 (VCAM-1), Endothelin 1 (ET-1), and von-Willebrand factor multimers (vWF), while nitrite and nitrate were measured as a nitric oxide metabolism end-product. To test the oxidative stress load on the graft at the time of reperfusion, the level of 8-hydroxy-2-deoxy Guanosine (8-OHdG), a bioproduct of DNA oxidation, was measured. To explore both clot formation and fibrinolysis during EVLP, Tissue Factor, Plasminogen Activator Inhibitor 1 (PAI-1), D-dimer, and platelet count were measured hourly. To evaluate the activation of the inflammasome, markers of both innate (procalcitonin, soluble Receptor of Advanced Glycation End-products (sRAGE), C-Reactive Protein, Pentraxin 3 (PTX-3), and Human High Mobility Group Protein B 1 (HMGB-1)) and cell mediated immunity (Regulated on Activation, Normal T Cell Expressed and Secreted (RANTES), Interleukin 6 (IL-6), Interleukin 8 (IL-8), Interleukin 1β (IL-1β), Interleukin 1 receptor antagonist (IL-1ra), Interleukin 10 (IL-10), Tumor Necrosis Factor α (TNF-α), and Phospholipase A2 Group VII (PLA2G7)) were measured.

Commercially available ELISA kits were used to assess IL-6, CXCL8/IL-8, IL-1ra, CCL5/RANTES, RAGE, TIMP-1, PTX-3, PAI-1, ET-1 (all reagents from R&D Systems INc),

and HMGB-1 (Nordic BioSite AB, Taby, Sweden). Further evaluations investigated the concentration of total hyaluronan (HA) (R&D Systems) and 8-hydroxy-2-deoxy Guanosine (8-OHdG) (DNA damage competitive ELISA kit, Thermo Fisher Scientific, Waltham, MA) in the perfusate.

The endogenous production of nitric oxide (NO) in the perfusates was evaluated through assessment of the total content of NO metabolites (nitrites and nitrates). A colorimetric commercially available kit with a detection range of 10-100 μM (Sigma-Aldrich) was used. Briefly, samples were loaded into Amicon Ultra 10 K centrifugal filter devices (Millipore, Burlington, MA, USA) and then subjected to 20 min of centrifugation at 7.000 x g (Haereus Multifuge X3R). Next, protein- and hemoglobin-free eluates were added to a nitrate reductase according to the manufacturer's instructions. Absorbance reading was performed at 540 nm (Synergy HTX, Biotek U.S).

Please see online supplement Table S2 for test sensitivity and standard curve values.

To account for perfusate substitution during the EVLP procedure, analyte concentrations were corrected according to the following formulae:

- T0: no correction
- T60 min: no correction

- T120 min: [(analyte concentration at T120 * total perfusate volume at T120) + (analyte concentration at T60 * removed volume at T60)]/ Steen total Volume

- T180 min: [(analyte concentration at T180 * total perfusate volume at T180) + (analyte concentration at T120 * removed volume at T120) + (analyte concentration at T60 * removed volume at T60)]/ Steen total Volume

- T240 min: [(analyte concentration at T240 * total perfusate volume at T240) + (analyte concentration at T180 * removed volume at T180)+ (analyte concentration at T120 * removed volume at T120)+ (analyte concentration at T60 * removed volume at T60)]/ Steen total Volume

<u>Supplement Results</u>

Supplement Figures Caption

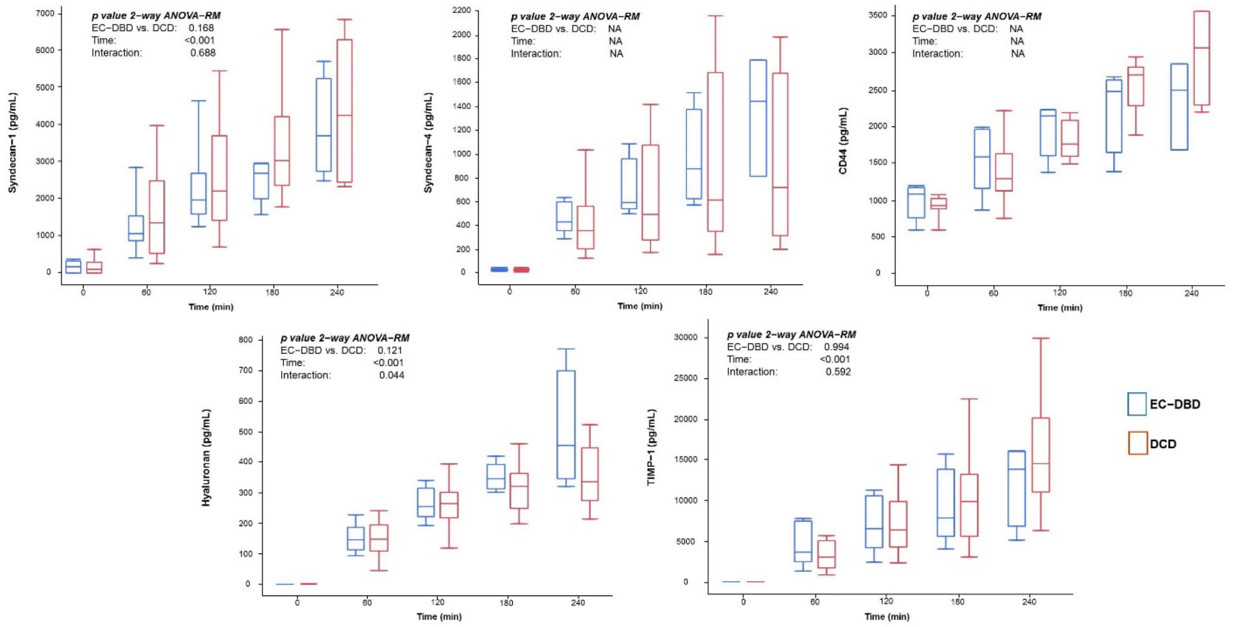

Figure S1. Glycocalyx Shedding.

Figure S1 illustrates the trend of glycocalyx shedding in the perfusate during the EVLP procedure. All molecule concentrations increased during the procedure, without relevant differences between the EC-DBD (blue) and DCD (red) grafts. Concentrations of tissue inhibitor of metalloproteinase (TIMP)-1 also did not differ between the two groups.

Abbreviations: EC-DBD, Extended-Criteria Donors after Brain Death; DCD, Donors after Cardiocirculatory Death; TIMP-1, Tissue Inhibitor Metalloproteinase-1; NA, Not Available.

Statistics: Two-Way ANOVA-RM, p value < 0.05 was assumed as statistically significant.

NA, sample not numerically acceptable for ANOVA-RM analysis.

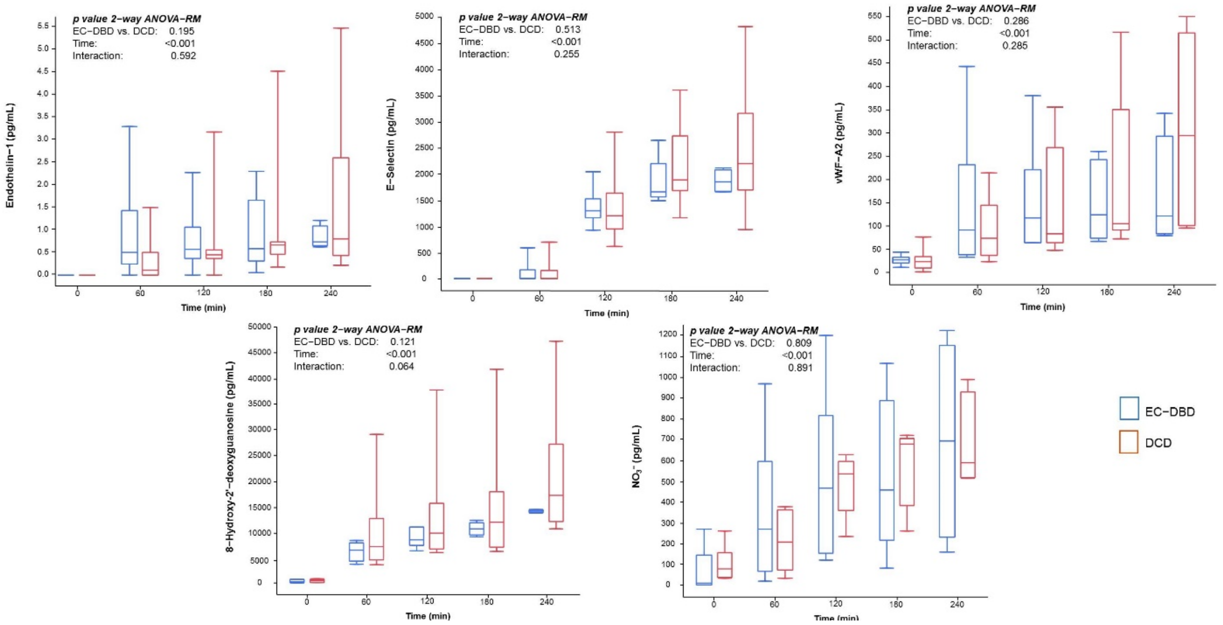

Figure S2. Endothelium Damage.

8-Hydroxy-2’deoxyguanosine, a marker of oxidative stress, reached a higher concentration in the perfusate of DCD grafts (red). Endothelin-1, Endothelial-Selectin and von Willebrand Factor A2 (vWF-A2), important markers of endothelial damage, were not

different between groups. The endothelial capacity to synthetize nitric oxide (NO), measured as total NO metabolites, appeared to be preserved in both groups.

Abbreviations: EC-DBD, Extended-Criteria Donors after Brain Death; DCD, Donors after Cardiocirculatory Death.

Statistics: Two-Way ANOVA-RM, p value < 0.05 was assumed as statistically significant

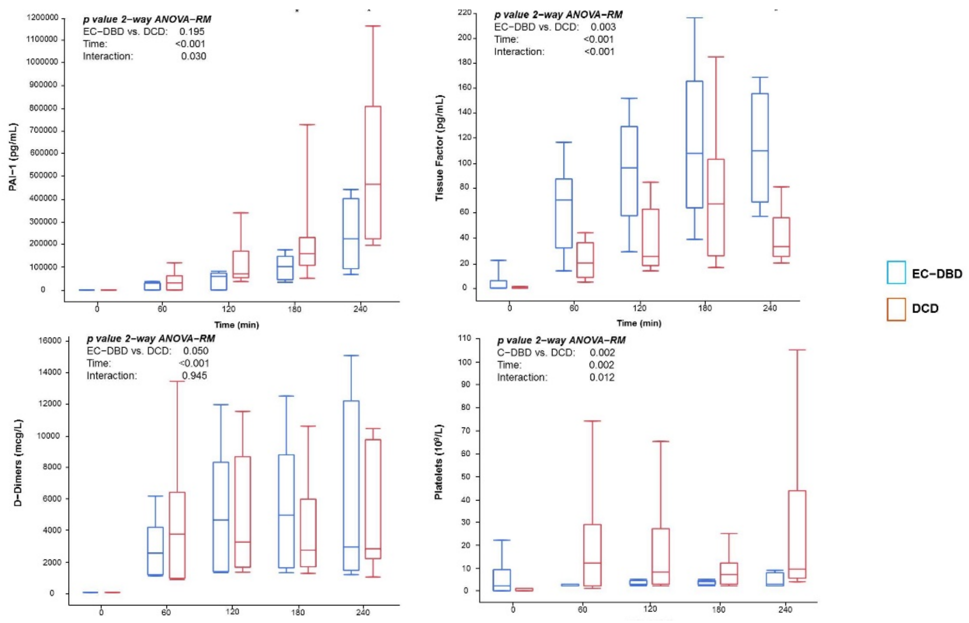

Figure S3. Coagulation Activation.

Higher platelet count, D-dimer, and plasminogen activator inhibitor 1 (PAI-1), along with reduced Tissue Factor in the perfusate of DCD grafts (red), were interpreted as activation of fibrinolysis leading to wash out of micro-thrombi with associated consumption of the main endovascular procoagulant factor.

Abbreviations: EC-DBD, Extended-Criteria Donors after Brain Death; DCD, Donors after Cardiocirculatory Death.

Statistics: Two-Way ANOVA-RM, p value < 0.05 was assumed as statistically significant

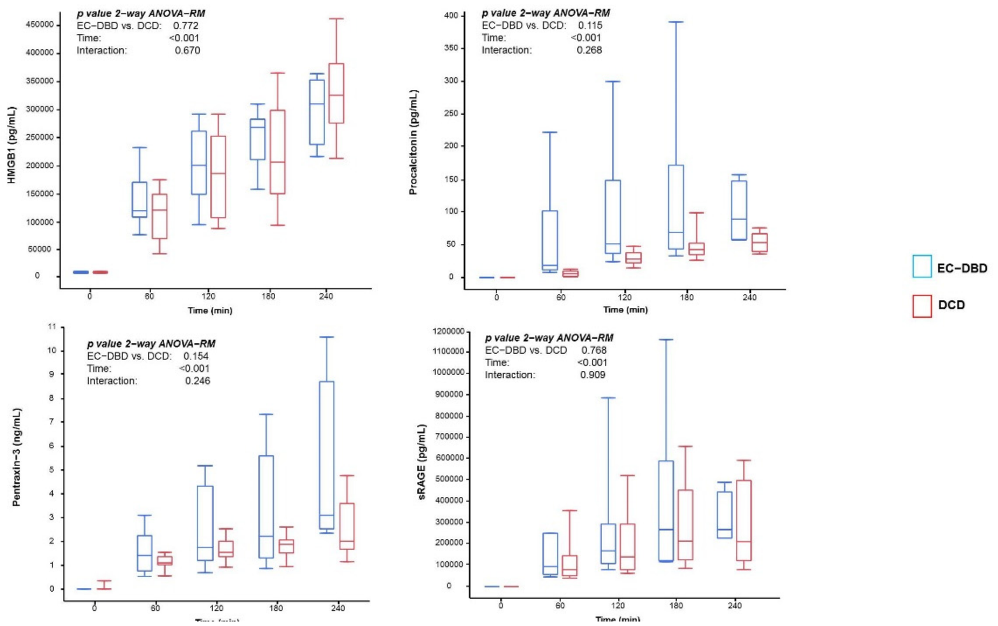

Figure S4. Innate Immunity Activation.

The High Mobility Group Box 1 (HMGB-1) – Receptor for Advanced Glycation Endproducts (RAGE) pathway was activated during EVLP, with no difference between EC-DBD (blue) and DCD (red) grafts. Procalcitonin and Pentraxin-3 showed a trend toward higher levels in the perfusate of DBD lungs.

Abbreviations: EC-DBD, Extended-Criteria Donors after Brain Death; DCD, Donors after Cardiocirculatory Death.

Statistics: Two-Way ANOVA-RM, p value < 0.05 was assumed as statistically significant.

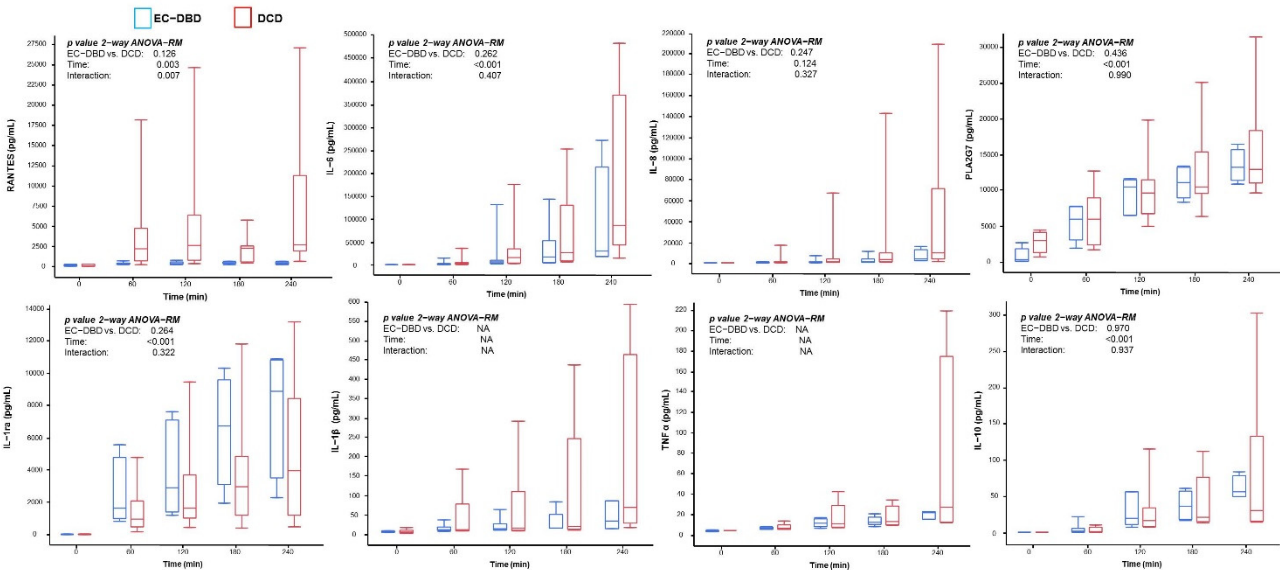

Figure S5. Inflammatory Mediators.

Despite the administration of high dose corticosteroids, significant production of pro-inflammatory mediators occurred during EVLP in both EC-DBD (blue) and DCD (red) grafts. Low levels of the anti-inflammatory cytokine IL-10 were measured in the perfusate of both groups.

Abbreviations: EC-DBD, Extended-Criteria Donors after Brain Death; DCD, Donors after Cardiocirculatory Death; RANTES, Regulated on Activation, Normal T cell Expressed and Secreted; IL, Interleukin; PLA2G7, Platelet-Activating Factor Acetylhydrolase; TNFα, Tumor Necrosis Factor Alpha; NA, Not Available

Statistics: Two-Way ANOVA-RM, p value < 0.05 was assumed as statistically significant.

NA, sample not numerically acceptable for ANOVA-RM analysis.

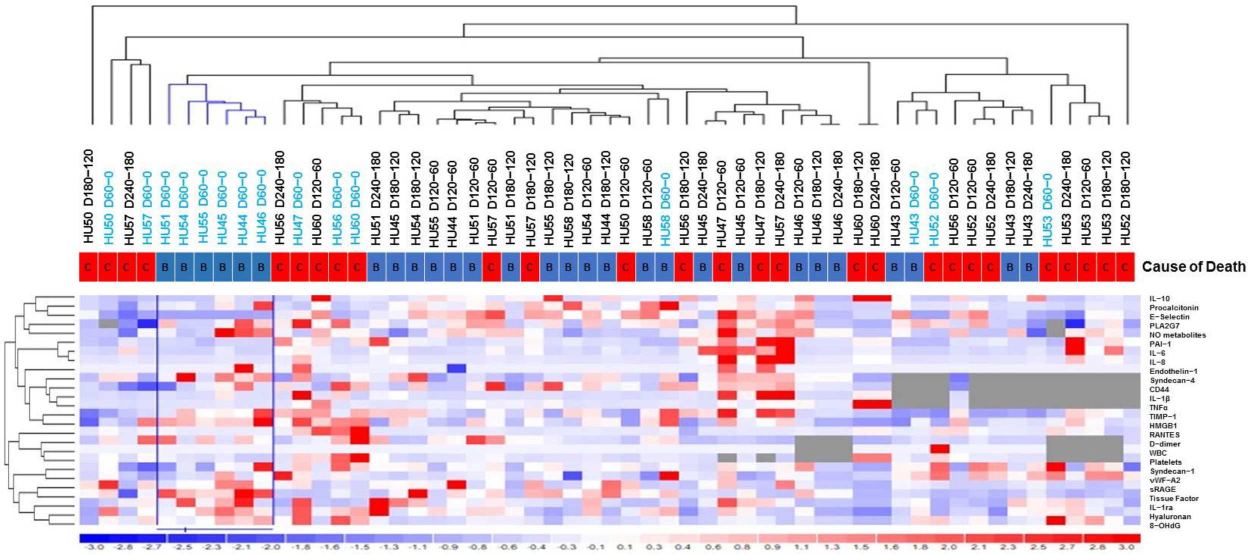

Figure S6. Unsupervised Cluster Analysis.

Cluster analysis was performed using dCHIP software (clustering method: average linkage; distance metric: 1–Spearman's rank correlation). Cause of death was listed as D for EC-DBD (**blue**) and C for DCD (**red**) donors. The columns identify the cases, while the rows denote the parameters evaluated in the perfusate. The degree of color saturation reflects the magnitude of the mediator concentration, as indicated in the color scale.

Supplement Tables

Table S1. Biomolecular Analysis: pathways and techniques.

Molecule	Pathway	Method
K^+, Na^{2+}, Cl^-, Ca^{2+}	Perfusate composition	Indirect ion-sensing
Albumin	Perfusate composition	Colorimetric assay
Hemoglobin	Perfusate composition	Colorimetric assay
ALT	Citolysis	Enzimatic assay
LDH<br>CPK		Colorimetric assay
Free Hemoglobin	Hemolysis	Colorimetric assay
Total Hyaluronan<br>CD44<br>Syndecan 1<br>Syndecan 4	Glycocalyx shedding	Luminex
TIMP-1		ELISA
Glucose<br>Lactate	Tissue Metabolism	Potenziometric Assay
BUN<br>Ammonium		Enzimatic assay
NO metabolites	Endothelial Damage	Colorimetric assay
E-Selectin<br>vWF		Luminex
ET-1<br>VCAM-1		ELISA
8-OHdG	Oxidative Stress	Competitive ELISA
Platelets count	Coagulation Activation	Automated cell counter
TF		Luminex
D-dimers		Immunoturbidimetric method
PAI1		ELISA
Procalcitonin	Innate Immunity	Luminex
CRP		Immunoturbidimetric method
PTX3<br>HMGB-1<br>sRAGE		ELISA
WBC count	Cell Mediated	Automated cell

	Immunity	counter
IL-1β<br>TNF-α<br>PLA2G7<br>IL-10		Luminex
RANTES<br>IL-6<br>IL-8<br>IL-1ra		ELISA

Abbreviations: ALT, Alanine Aminotransferase; BUN, Blood Urea Nitrogen; Ca^{2+}, Calcium Ion; Cl^{-}, Chlorine Ion; CPK, Creatin Phosphokinase; CRP, C-Reactive Protein; ELISA, Enzyme-Linked Immunosorbent Assay; E-Selectin, Endothelial-Selectin; ET-1, Endothelin 1, HMGB-1: Human High Mobility Group Protein B 1; K^{+}, Potassium Ion; IL-1β, Interleukin 1β; IL-6, Interleukin 6; IL-8, Interleukin 8; IL-1ra, Interleukin 1 Receptor Antagonist; IL-10, Interleukin 10; LDH, Lactate Dehydrogenase; Luminex, Human Magnetic Luminex Screening Assay; Na^{2+}, Sodium Ion; NO metabolites, Nitric Oxide Metabolites (Nitrite and Nitrate); 8-OHdG, 8-hydroxy-2-deoxy Guanosine; PAI1, Plasminogen Activator Inhibitor 1; PLA2G7, Phospholipase A2 Group VII; PTX3, Pentraxin 3; RANTES, Regulated on Activation, Normal T Cell Expressed and Secreted; sRAGE, soluble Receptor of Advanced Glycation End-products; TF, Tissue Factor; TIMP-1, Tissue Inhibitor of Metalloproteinase 1; TNF-α, Tumor Necrosis Factor α; VCAM-1, Vascular Cell Adhesion Molecule 1; vWF, von-Willebrand Factor Multimers; WBC, White Blood Cell.

Table S2. Biomolecular Analysis Assay.

Assay	Method	Assay range	Sensitivity	Units	Company
Coagulation Factor III /Tissue Factor	Human Magnetic Luminex® Screening Assay*	6-1490		pg/ml	
Procalcitonin	Human Magnetic Luminex® Screening Assay*	9-2110		pg/ml	
TNF-α	Human Magnetic Luminex® Screening Assay*	90-2240		pg/ml	
CD44	Human Magnetic Luminex® Screening Assay*	107-25980		pg/ml	
PLA2G7/PAF-AH/Lp-PLA2	Human Magnetic Luminex® Screening Assay*	3331-809380	Not Specified	pg/ml	R&D Systems
vWF-A2	Human Magnetic Luminex® Screening Assay*	40-9700		pg/ml	
E-Selectin /CD62E	Human Magnetic Luminex® Screening Assay*	330-80300		pg/ml	
Syndecan-1/CD138 ELISA	Human Magnetic Luminex® Screening Assay*	125-30460		pg/ml	
Syndecan-4	Human Magnetic Luminex® Screening Assay*	57-13780		pg/ml	
IL-1β/IL-1F2	Human Magnetic Luminex® Screening Assay*	17-4180		pg/ml	
IL-10	Human Magnetic Luminex® Screening Assay*	4-880		pg/ml	
Human TIMP-1 Quantikine ELISA kit	Enzyme -Linked Immunosorbent Assay (ELISA)	156-10000	80	pg/ml	
Human IL-1ra/IL-1F3 Quantikine ELISA kit	Enzyme-Linked Immunosorbent Assay (ELISA)	31.2-2000	18.3	pg/ml	
Human IL-6 Quantikine ELISA kit	Enzyme-Linked Immunosorbent Assay (ELISA)	3.1-300	0.7	pg/ml	
Human IL-8/CXCL8 Quantikine ELISA kit	Enzyme-Linked Immunosorbent Assay (ELISA)	31.2-200	7.5	pg/ml	R&D Systems
Human sVCAM-1/CD106 Quantikine ELISA kit	Enzyme-Linked Immunosorbent Assay (ELISA)	6250-200000	1260	pg/ml	
Human CCL5/RANTES Quantikine ELISA kit	Enzyme-Linked Immunosorbent Assay (ELISA)	31.2-200	6.6	pg/ml	
Human Serpin E1/PAI-1 Quantikine ELISA kit	Enzyme-Linked Immunosorbent Assay (ELISA)	313-20000	142	pg/ml	
Human Pentraxin 3/TSG-14 Quantikine ELISA kit	Enzyme-Linked Immunosorbent Assay (ELISA)	0.3-20	0.116	ng/ml	

Human RAGE Quantikine ELISA kit	Enzyme-Linked Immunosorbent Assay (ELISA)	78-2000	16.4	pg/ml	
Endothelin-1 Quantikine ELISA kit	Enzyme-Linked Immunosorbent Assay (ELISA)	0.4-25	0.207	pg/ml	
Hyaluronan Quantkine ELISA kit	Enzyme-Linked Immunosorbent Assay (ELISA)	0.6-40	0.2	ng/ml	
Human HMGB1 (High Mobility Group Protein B1)	Enzyme-Linked Immunosorbent Assay (ELISA)	31.3-2000	18.75	pg/ml	Nordic BioSite
DNA Damage Competitive ELISA Kit	Competitive Enzyme-Linked Immunosorbent Assay (ELISA)	62.6-8000	50.9	pg/ml	Thermo Fisher
Nitrite/Nitrate Assay Kit	Colorimetric	0-6201	Not Specified	ng/ml	Sigma-Aldrich

*Human Premixed Multi-Analyte Kit.

Table S3. Pearson Correlation analysis between donor warm ischemia time and biomolecular profile.

	rho	R^2	p value
**DWIT/sRAGE**	-0.139	0.0193	0.766
**DWIT/PAI-1**	0.789	0.6225	0.035
**DWIT/TIMP-1**	0.890	0.7919	0.008
**DWIT/IL-1ra**	0.495	0.2446	0.259
**DWIT/Endothelin 1**	0.700	0.4904	0.080
**DWIT/RANTES**	0.187	0.0351	0.688
**DWIT/Hyaluronan**	0.316	0.0997	0.490
**DWIT/8-Hydroxy-2'-deoxyguanosine**	0.0790	0.0062	0.866
**DWIT/IL-6**	0.518	0.2685	0.234
**DWIT/CXCL8/IL-8**	0.702	0.4931	0.079
**DWIT/NO_3^-**	0.639	0.4085	0.122
**DWIT/Pentraxin-3**	0.245	0.0602	0.596
**DWIT/HMGB1**	0.841	0.7065	0.018
**DWIT/Tissue Factor**	-0.495	0.2446	0.259
**DWIT/IL-10**	0.232	0.0539	0.616
**DWIT/Procalcitonin**	0.177	0.0312	0.705
**DWIT/PLA2G7**	0.851	0.7241	0.015
**DWIT/Syndecan-1**	0.102	0.0103	0.828
**DWIT/vWF-A2**	0.095	0.0092	0.838

IL-1β, Syndecan-4, CD44, and TNF-α are not present in the Table because the sample size was not sufficient for Pearson correlation analysis.
